# Extracellular Vesicles and Cancer: A Focus on Metabolism, Cytokines, and Immunity

**DOI:** 10.3390/cancers12010171

**Published:** 2020-01-10

**Authors:** Donatella Lucchetti, Claudio Ricciardi Tenore, Filomena Colella, Alessandro Sgambato

**Affiliations:** 1 Fondazione Policlinico Universitario Agostino Gemelli IRCCS, 00168 Roma, Italy; dnlucchetti@gmail.com; 2 Institute of General Pathology, Catholic University of the Sacred Heart, 00168 Roma, Italy; c.ricciarditenore@hotmail.it (C.R.T.); colella.filomena@gmail.com (F.C.); 3 Centro di Riferimento Oncologico della Basilicata (IRCCS-CROB), Rionero in Vulture, 85028 Potenza, Italy

**Keywords:** extracellular vesicles, immune cells, cytokines, metabolism, tumor microenvironment

## Abstract

A better understanding of the mechanisms of cell communication between cancer cells and the tumor microenvironment is crucial to develop personalized therapies. It has been known for a while that cancer cells are metabolically distinct from other non-transformed cells. This metabolic phenotype is not peculiar to cancer cells but reflects the characteristics of the tumor microenvironment. Recently, it has been shown that extracellular vesicles are involved in the metabolic switch occurring in cancer and tumor-stroma cells. Moreover, in an immune system, the metabolic programs of different cell subsets are distinctly associated with their immunological function, and extracellular vesicles could be a key factor in the shift of cell fate modulating cancer immunity. Indeed, during tumor progression, tumor-associated immune cells and fibroblasts acquire a tumor-supportive and anti-inflammatory phenotype due to their interaction with tumor cells and several findings suggest a role of extracellular vesicles in this phenomenon. This review aims to collect all the available evidence so far obtained on the role of extracellular vesicles in the modulation of cell metabolism and immunity. Moreover, we discuss the possibility for extracellular vesicles of being involved in drug resistance mechanisms, cancer progression and metastasis by inducing immune-metabolic effects on surrounding cells.

## 1. Introduction

Cancer cells heterogeneity has a strong impact on tumor progression and metastasis, and tumor-associated stromal cells are a key player in this phenomenon.

Cooperative cancer cell interaction with surrounding cells is mediated by several mechanisms of intercellular communication, including secretion of growth factors, cytokines and chemokines, and the production and release of extracellular vesicles (EVs). EVs are a heterogeneous group of cell-derived membranous organelles, which allows cells to exchange proteins, lipids and genetic material and to influence the behavior of recipient cells. Although Wolf and colleagues initially considered EVs only as waste released by cells, growing evidence in the field has highlighted their role as signaling messengers in physiological and pathological processes, including cancer development [1]. Based on their biogenesis, EVs can be divided into two main categories comprising exosomes, which originate within the endosomal system, and microvesicles, that are shed from the plasma membrane. Based on their size (and on their current method of isolation regardless of their biogenesis), EVs can be grouped as follows: medium extracellular vesicles (mEVs, with a size of 150–1000 nm), small extracellular vesicles (sEVs, 40–150 nm), apoptotic vesicles (ApoEVs, 100–1000 nm), and apoptotic bodies (1000–5000 nm). In this manuscript, we refer to sEVs and mEVS following the guidelines of ISEV (International Society for Extracellular Vesicles) with some modification [2,3,4]. When size is not specified, we used the generic term of EVs.

This paper reviews the available evidence on the metabolism of cancer and tumor-associated stromal cells and the roles of immune cells in the tumorigenic process focusing on EVs.

## 2. Metabolism of Cancer Cells

Metabolism represents the totality of reactions that produce energy for maintaining the cells alive. It is a balance between anabolism (building up) and catabolism (breakdown), resulting in the generation of chemical energy (ATP) essential for cell activities. Metabolism is also important for the production of intermediates consumed in the anabolic reactions and for the generation of metabolites used in enzymatic reactions [5].

In contrast to normal cells, cancer cells require a massive amount of glucose to achieve their biosynthetic and bioenergetics needs by uncoupling glycolysis from the TCA (tricarboxylic acid) cycle (also known as Krebs cycle). This metabolic phenomenon is referred to as “aerobic glycolysis” or the “Warburg effect” [6]. Briefly, cancer cells metabolize glucose to pyruvate through glycolysis and, even in aerobic conditions, most pyruvate is converted to lactate in the cytoplasm by the action of lactate dehydrogenase (LDH) and released into the tumor microenvironment (TME) [7]. Moreover, cancer cells which are in poorly oxygenated microenvironments are forced to activate glycolysis and to secrete lactate. Lactate is not used as a waste product but internalized by other tumor cells that are in normoxic condition (near to blood vessel) and used as an alternative energy source by conversion into pyruvate, which then fuels the TCA cycle [8,9]. In the meantime, the TCA cycle is also replenished by an increased consumption of glutamine [10,11]. Noteworthy, the PI3K/AKT/mTOR signaling pathway drives the Warburg effect in cancer cells. Protein kinase B (PKB), also known as AKT, the main effector of PI3K, induces glucose uptake, mediated by glucose transporters GLUT1 and GLUT4 [12], and increases glucose metabolism by phosphorylating hexokinase 2 [13] and indirectly activates PFKFB2, which generates fructose 2,6-bisphosphate that activates phosphofructokinase-1, one of the most important regulatory enzymes of glycolysis [14].

Glycolysis rapidly synthesizes two moles of ATP per mole of glucose, up to 100 times faster than oxidative phosphorylation (OXPHOS), whereas OXPHOS generates up to 36 ATPs per mole of glucose [15]. To balance the yield and rate of ATP production, the tumor microenvironment is characterized by metabolic heterogeneity: some cancer cells exploit the glycolytic metabolism and others the OXPHOS [15]. Unlike what was believed so far, it has been recently demonstrated that many tumors are highly dependent on OXPHOS for ATP synthesis, and Molina and colleagues showed that a subset of tumors depends on OXPHOS not only for ATP but also for nucleotide biosynthesis [16]. Interesting experimental data showed that the loss of OXPHOS does not allow the formation of tumors in vivo [17]. Lung cancer cells with genetic mutation in SWI/SNF complex or KRAS oncogene are dependent on OXPHOS for their growth and survival [18,19]. This feature has been also reported in glioblastomas with mutation of enolase [20]. For example, genetic deletion of the KRAS^G12D^ allele leads to a dramatic tumor regression but some pancreatic cancer cells are resistant and can cause tumor relapse: it has been seen that these cancer cells are OXPHOS dependent [21]. Thus, OXPHOS inhibitors have been suggested as anticancer drugs and some of them are currently being evaluated in phase 1 clinical trials [22]. Very recently, Fisher and colleagues reported that in melanoma brain metastases, the OXPHOS is more strongly utilized compared to extracranial metastases and primary melanomas [23].

## 3. Metabolic Relationship between Cancer Cells and Tumor-Associated Stromal Cells

The tumor microenvironment (TME) comprises a heterogeneous population of cancer cells and nearby stromal cells recruited by the tumor (tumor-associated stromal cells, TASCs) [24]. Stromal cells include fibroblasts, adipocytes, endothelial cells, bone-marrow mesenchymal stromal cells (MSCs), and immune cells. These cells promote events such as proliferation, extracellular matrix remodeling, cellular motility, neo-vascularization, tumorigenesis, drug resistance, and tumor evasion of immunosurveillance through the production of various chemokines, growth factors, and cytokines [25].

Evidence shows that fibroblasts represent most of the stromal cells within a tumor [26] and cancer-associated fibroblasts (CAFs) can coevolve with cancer, transitioning from an inactivated to an activated state [27,28]. During tumor progression, fibroblasts increase proliferation, promote tumor growth, and mediate therapeutic resistance [29]. CAFs, unlike normal fibroblasts (NF), have an active behavior: they are similar to myofibroblasts, responsible for wound healing and chronic inflammation, and are characterized by elevated expression of α-smooth muscle actin (α-SMA) [30,31]. CAFs can also display an increase in aerobic glycolysis and this process has been referred to as the reverse Warburg effect to distinguish the CAF-related phenomenon from the cancer cells counterpart [32]. Pavlides showed that the loss of Caveolin-1 can confer to stromal cells a cancer-associated fibroblast phenotype; these authors showed that 25 proteins were overexpressed and eight of them play a crucial role in the Warburg effect [30,31,33,34] while two, peroxiredoxin 1 and catalase, are involved in oxidative stress [32]. Shan et al. provided further evidence to support the reverse Warburg hypothesis: pancreatic-associated fibroblasts are responsible for lactate secretion by the expression of glycolytic enzymes LDHA (lactate dehydrogenase A) and PKM2, as well as the MCT4 (monocarboxylate transporter 4) transporter. It has also been observed that an observable enlargement of the mitochondria and an enhanced aerobic activity was evident when pancreatic cancer cells were exposed to CAFs conditioned media [35]. Moreover, pancreatic cancer cells significantly increased expression of MCT1 (monocarboxylate transporter 1), FH (fumarate hydratase), and SDH (succinate dehydrogenase), showing the existence of metabolic cooperation between CAFs and cancer cells [35]. Apicella and colleagues reported that in lung cancer and gastric cancer cells derived from patient tumors treated with tyrosine kinase inhibitors, there is an increase of glycolytic metabolism and lactate release that act as molecule driving CAFs to secrete more hepatocyte growth factor (HGF), which overcomes TKI inhibitory effects [36].

Overall, the evidence suggests that a reciprocal metabolic relationship exists between CAFs and cancer cells that is essential for cancer progression.

Cancer-associated adipocytes (CAAs) represent another important component of TME and they contribute to extracellular matrix remodeling, invasion, and survival of cancer cells, and epithelial to mesenchymal transition (EMT) [37]. In particular, Wang et al. discovered that CAAs, compared with their normal adipocytes counterpart, produce increased amounts of insulin-like growth factor binding protein-2 (IGFBP-2) which promotes cancer cell migration and metastasis. The expression of matrix metalloproteinases (MMPs) was increased by co-culturing mature adipocytes with cancer cells, improving their invasive properties [38,39]. A similar phenomenon during the adipocyte and cancer cell co-culture was observed for the pro-inflammatory cytokines IL-6, IL-1β, IL-8, and for the fatty acid-binding proteins (FABPs), which was found to promote homing, migration, and invasion of cancer cells [40]. Nieman et al. suggested that CAAs might represent an additional source of energy for cancer cells. In fact, they showed that lipolysis in the adipocytes is stimulated by the contact of adipocytes with cancer cells. Adipocytes promote the growth of cancer cells by providing energy-dense lipids supporting their rapid growth (Table 1). Adipokines (leptin, adiponectin, autotaxin, interleukin 6, TNFα, and HGF) secreted by adipocytes increase the mitochondrial β-oxidation in breast cancer cells. Moreover, CCAs release a significant number of metabolic substrates that are used by cancer cells to build complex macromolecules: glycerol and fatty acid to build complex lipids for membrane synthesis, amino acids for protein synthesis, and nucleotides for DNA and RNA synthesis [41].

The metabolism of cancer cells and immune cells in TME is an important weapon for tumor initiation, progression, and metastasis. Differentiation and activation of pro-tumor immune cells, including myeloid-derived suppressor cells (MDSCs), M2 macrophages, regulatory T cells, and suppressing antitumor immune cells (CD8+ T cells, M1 macrophages, and N1 neutrophils) are promoted by the deprivation of nutrients [42]. Tumor-associated immune cells regulate their metabolism for survival, differentiation, and pro/antitumor functions by modulating several signaling pathways, such as PI3K-Akt, mTOR, HIF-1, and c-Myc [42]. Glycolytic metabolism activated by inflammation may be upregulated during the tumor-initiation process by HIF-1 activation in both cancer and immune cells. Particularly, during tumor progression, HIF-1 up-regulation in tumor-associated macrophages (TAMs), tumor-associated dendritic cells (TADCs), MDSCs, and Tregs contributes to immune suppression and angiogenesis by PD-L1 expression, adenosine-adenosine receptor interaction, and lactate release that facilitate tumor growth [42].

## 4. Extracellular Vesicles and Cancer

**Figure 1:**
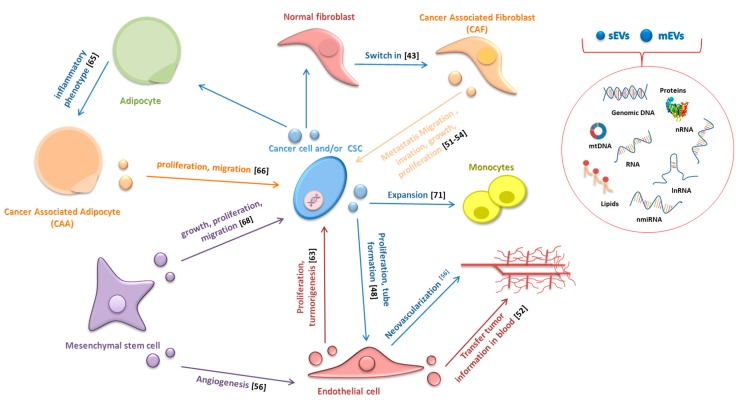
In tumor microenvironments, EVs transport information from cancer cells to other cells and vice versa, and represent an important mechanism of intercellular communication.

Extracellular vesicles (EVs) play a key role in tumor growth, invasion, and metastasis, transferring horizontally several surface markers, signaling molecules, oncogenic proteins, and nucleic acids to stromal cells, thus modifying their behavior. Indeed, several studies have shown that EVs can affect the behavior of CAFs, endothelial cells, CAAs, mesenchymal stem cells (MSC), and immune cells (the relationship between EVs and cells of the immune system will be discussed in detail in Section 6; Figure 1). Ten years ago, Webber and colleagues showed that sEVs released by cancer cells could transmit information to normal stromal fibroblasts (NFs) and trigger a cellular response, which would entail a differentiation of NFs to a myofibroblast phenotype, and they indicated TGFβ as the molecule mainly responsible for this differentiation [43]. When associated with sEVs, TGFβ induces the transcription of mRNAs similar but not identical to those induced by TGFβ in soluble form; particularly, mesothelioma-released sEVs stimulated a strong induction of FGF2 mRNA while TGF-β treatment modestly induced FGF2 expression [43].

Afterwards, several studies have highlighted the involvement of various microRNAs transferred by EVs in the switch of NFs to CAFs (Table 2). However, the relationship is reciprocal: not only cancer-derived EVs can promote changes in tumor stromal cells but CAFs-derived EVs can also, on the other hand, increase cancer growth, progression, and metastasis. Luga and colleagues showed that CAFs-secreted sEVs are potent regulators of the Wnt-planar cell polarity pathway in breast cancer cells favoring cancer metastasis [44] and Santi demonstrated that CAFs transfer lipids and proteins to cancer cells through EVs supporting tumor growth [45]. Recently, Dourado and colleagues suggest that EVs released by CAFs induce migration and invasion of oral squamous cell carcinoma cells [46]. Moreover, Qin X and colleagues provided evidence that EVs contain miR-196a, which confers cis-platin resistance in head and neck cancer through targeting CDKN1B and ING5 [47]. EVs also play a key role in cancer-associated angiogenesis by stimulating the proliferation of endothelial cells, tube formation, and neovascularization [48]. Grange and colleagues demonstrated that mEVs released from human renal cancer stem cells stimulate angiogenesis, promote metastatic niche, and greatly enhance lung metastases in SCID mice following i.v. injections of renal carcinoma cells [48]. EVs have also been shown to transfer several angiogenic factors, such as CD47, CD147, Egr-1, miR135b, Rak1, and PAK2, to endothelial recipient cells thus confirming their pro-angiogenic ability [49,50,51,52,53]. McCann J.V. and colleagues demonstrated that endothelial cells-derived EVs (EC-EVs) transfer information to different cell types present in primary tumors and sites of metastasis when secreted in the systemic circulation [54]. For example, glioma-associated human endothelial cells, in addition to secreting cytokines, provide a tumor supportive microenvironment by releasing EVs enriched in CD9. In this manner, they influence recipient glioma cancer stem cells, promoting proliferation, self-renewal and tumor-sphere formation in vitro and tumorigenicity in vivo [55].

CAAs-EVs can also contribute to drive cancer cells towards a more aggressive phenotype through different mechanisms: (1) the transfer of enzymes implicated in fatty acid oxidation; (2) increasing MMP9 activity via transferring MMP3, as described in lung cancer cells; and (3) activation of Hippo signaling pathway, as observed in breast cancer cells [64]. Wang S. and colleagues demonstrated that sEVS released by hepatocellular carcinoma cells are incorporated by adipocytes and induce an inflammatory adipocyte phenotype [65]. Moreover, they showed that sEVS released by MSC differentiated in adipocytes and are internalized by breast cancer cells promoting their proliferation and migration through the activation of the HIPPO signaling pathway [66].

Despite the scientific interest toward CAAs-EVs, further studies on adipose-derived EVs are needed to fully understand their roles in the process of cancer development.

The interest in the role of EVs in cancer metastasis began with Peinado’s study: sEVs from highly metastatic melanomas increased the metastatic behavior of primary tumors by reprogramming the bone marrow progenitors-derived cells through the MET tyrosine kinase receptor [67]. Other studies demonstrated the involvement of MSCs-derived EVs in supporting tumor growth, increasing migration, participating in the acquisition of apoptosis resistance, and stimulating angiogenesis [68]. Hoshino et al. reported very interesting results: sEVs released from different types of cancer cells were preferentially up taken by organ-specific sites of metastasis based on sEVs integrins expression patterns [69].

EVs also play a key role in cell stress response, resulting in a transfer of a resistant phenotype and immune evasion [70]. In support of this evidence, it has been recently demonstrated that EVs positive for annexin-A6, released by cancer cells following chemotherapy, promote NF-κB-dependent endothelial cell activation, Ly6C+CCR2+ monocyte expansion, and MCP-1 induction to facilitate the establishment of breast cancer lung metastases [71]. Pavlyuko et al. showed that Apo-EVs produced by cancer cells undergoing stress, by irradiation, or temozolomide/cisplatin administration, promote malignancy of glioblastoma cells by transferring various components of spliceosomes [71,72]. Overall, the available data suggest that EVs play important roles in the tumorigenic process, being involved in tumor progression, tumor-associated inflammation as well as tumor niche remodeling and metastasis. Indeed, the information carried by EVs (proteins, lipids, mRNA, miRNA, lnRNA, DNA) can reprogram the recipient cells, modulating their phenotype and their behavior [73]. Specifically, cancer cell-derived EVs mediate the transfer of information between different cancer cells but can also have a significant impact on the function and behavior of non-cancerous cells within TME. On the other hand, tumor-stroma cells can affect cancer cells and contribute to tumor initiation and progression [74,75,76,77].

## 5. Role of Extracellular Vesicles in Cancer Cells Metabolism

**Figure 2:**
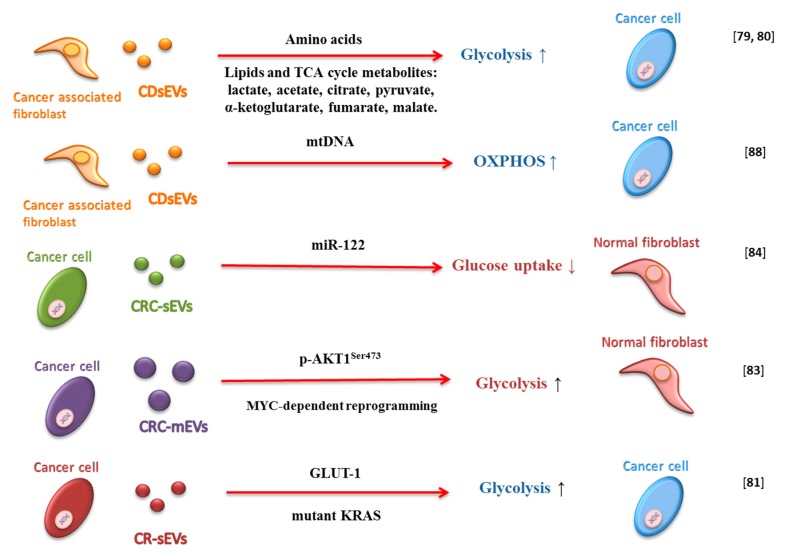
Schematic representation of how extracellular vesicles (Evs) influence metabolism in the tumor microenvironment. Small extracellular vesicles derived from cancer-associated fibroblast (CAF-EVs) can stimulate glycolysis and OXPHOS by metabolite cargo and mtDNA. Colorectal cancer-derived small extracellular vesicles (CRC-sEVS) can transport miRNA (i.e., miR-122) that can reduce glucose uptake in normal fibroblasts, and other proteins and enzymes that influence glycolysis in cancer cells. Finally, colorectal cancer-derived medium extracellular vesicles (CRC-mEVs) can increase glycolysis in normal fibroblasts.

CAFs are the major constituents of TME in many cancers and can induce metabolic reprogramming of cancer cells (Figure 2) [78]. Zhao and colleagues demonstrated that CAF-derived sEVs (CAF-EVs) modulate cancer cells metabolism in prostate and pancreatic cancer [79]. They observed that CAF-EVs metabolite cargo, including amino acids, acetate, stearate, palmitate, and lactate, could shift cellular metabolism towards glycolysis in prostate cancer cells [79]. Additionally, the metabolite cargo of CAF-EVs rescues the proliferation of pancreatic ductal adenocarcinoma (PDAC) cells under nutrient-deprived stress conditions [80]. Achreja and colleagues also demonstrated that cancer cells internalize CAF-EVs rapidly and that this phenomenon influences intracellular metabolism. Moreover, CAF-EVs, by providing lactate, regulate glycolysis fluxes and contribute up to 35% of the TCA cycle fluxes by supply TCA intermediates and glutamine [80].

sEVs derived from colorectal cancer (CRC) mutant KRAS-expressing cells contain proteins and enzymes involved in metabolism and glycolysis and can enhance the growth of wild type KRAS cells [81]. Indeed, Zhang et al. showed that these sEVs are able to confer a Warburg-like effect on recipient colonic epithelial cells in vitro and in vivo. They observed that sEV-GLUT-1 is responsible for increased cellular glucose uptake, and consequently, increased aerobic glycolysis in recipient tumor cells [82].

It has also been demonstrated that mEVs isolated from plasma of patients with metastatic prostate cancer promote MYC-dependent reprogramming of human normal prostate fibroblasts in an AKT1-dependent manner. Moreover, such mEVs are able to upregulate molecules involved in stroma activation and angiogenesis and can contribute to tumor progression by altering the metabolism of the target fibroblasts. In fact, they can induce glutaminase and LDH (lactate dehydrogenase) upregulation and a significant correlation between LDH and MYC has been reported in the tumor stroma [83].

microRNAs and small nuclear RNAs are packaged in EVs and can contribute to regulate the biology of recipient cells [84,85,86]. Fong and colleagues demonstrated thatEVs with high levels of miR-122 secreted by breast cancer cells can influence fibroblasts metabolism. The miR-122 transferred by EVs reduces glucose uptake in the surrounding normal cells through downregulation of PKM2 and GLUT1. In this manner, glucose availability for cancer cells increases, leading to enhanced cell proliferation and promoting metastasis [84].

EVs do not just transfer genomic DNA from one cell type to another [87] but also to mtDNA. Sansone and colleagues observed the full mitochondrial genome packaged in CAF-derived sEVs and sEVs isolated from patients with hormonal therapy-resistant breast metastatic disease. The acquisition of CAF-derived EVs-mtDNA by breast cancer cells influence cell metabolism, promoting estrogen receptor-independent oxidative phosphorylation and mediating exit from therapy-induced metabolic dormancy (Figure 2) [88].

## 6. Immunity and Extracellular Vesicles in Cancer

The host’s immune response often uses EVs to perform its function. For example, dendritic cell-derived sEVs are crucial in the activation of immune response against tumors and in the cytolytic activity of natural killer (NK) cells [89,90].

On the other hand, it has been reported that tumor-derived EVs have an immunosuppressive effect on peripheral blood mononuclear cells (PBMCs) at high concentrations (up to 100 μg/mL) [91]. Cancers can adopt several EV-based approaches to interfere with the immune system. Tumor-derived EVs mediate an immunosuppression activity during tumor progression by (1) suppression of immune effector cells, (2) exchange of nucleic acids, and (3) changing the recipient cells’ transcriptome profile. On the contrary, it has been shown a stimulatory effect of EVs on immune cells carrying the tumor-associated antigens costimulatory molecules and major histocompatibility complexes (MHC) components [92]. Tumor-derived EVs can interact and affect the behavior of immune cells through receptor-ligand binding interaction or by internalization [92]. Tumor-derived EVs can also interact with lymphocytes binding to cellular MHC receptors through ligands or antigens exposed on their membrane or carried by them, thus altering immune function [93]. In addition, phagocytic cells, such as macrophages and dendritic cells, can easily uptake tumor-derived EVs. Moreover, EVs released by tumor cells, containing so-called death ligands (such as Fas ligands or TNF-α), have the potential to directly induce cell death in immune cells binding the death receptor family members TNF receptor 1 (TNFR1) and Fas receptor (FasR), which induce necrosis or apoptosis, respectively [89].

Tumors can escape from cytotoxic T-lymphocytes (CTL) detection in EV-mediated manner: TGF-β on the surface of EVs function to suppress CTL activity. Moreover, to escape from the attack of NK cells, tumors can release EVs interfering with their cytotoxic activity [91]. EVs bearing ligands of the NK cell-activating receptor NK group 2 member D (NKG2D) act as bait for NK cells thus distracting immune cells from the tumor [89,94]. Additionally, in some cases, these EVs elicit a downregulation of NKG2Don natural killer (NK) and CD8+ T cells [95,96].

Much evidence has shown that cancer cells adapt to a hypoxic microenvironment for their survival [97]. Berchem and colleagues showed how EVs released by cancer cells upon hypoxic conditions had a stronger inhibitory impact on NK cells compared to those producing from normoxic conditions. The transfer of miR-23a and TGF-β to NK cells could be attributed to an increase of immunosuppressive potential [98]. Ding and colleagues showed an inhibition of mRNAs expression and an increase of cancer-related miRNAs in DCs treated with pancreatic cancer-derived EVs [99]. In conclusion, the transfer of inhibitory miRNAs or mRNAs by tumor derived-EVs can promote cancer progression by negatively influencing the host’s immune response in different cancer types [100,101].

Cancer cell-derived sEVs harboring PD-L1 can inhibit T cell functions, thus promoting tumor growth as demonstrated by two different studies: the first shows how breast cancer cell-derived sEVs can transfer PD-L1 to other cancer cells and block T cell activity through interaction with PD1 [102]; the second one reported that sEVs from human lung cancer, melanoma, or breast cancer express PD-L1 on their surface and that monitoring these circulating sEVs can be used to predict patient response to anti-PD1 therapy [103].

Noteworthy is the article by Li et al. which investigates the role of tumor-derived sEVs in mediating the anti- and pro-tumoral equilibrium of γδ T cells in normoxic and hypoxic conditions. γδ T cells are a unique lymphocyte population reported to have either anti- or pro-tumoral functions in several cancer types [104]. γδ T cells represent a 0.5%–16% of total CD3+ cells in the circulation, and are present mostly in intestine and skin [105].

γδ T can carry out a pro-tumoral role by secreting tumor necrosis factor (TNF), IL-8, and granulocyte-macrophage colony-stimulating factor, inducing an accumulation of immunosuppressive myeloid derived suppressor cells (MDSCs) driving pro-tumoral inflammation [106]. A hypoxic microenvironment increases the release of miR-21-rich sEVs that may transfer to normoxic regions and drive non hypoxic cells toward a prometastatic phenotype and activate the γδ T-cell expansion [107]. Normoxic tumor-derived EVs administration on γδ T cells induced their expansion and cytotoxicity against oral squamous cell carcinoma. Nevertheless, the effects of normoxic tumor-derived EVs were attenuated by hypoxic tumor EVs, which enhanced the suppressive role of MDSCs in a miR-21/phosphatase and tensin homolog (PTEN)/programmed death ligand-1 (PD-L1)-axis-dependent manner [104].

Cancer-derived sEVs can inhibit DC activity and induce an expansion of MDSCs. Zhou and colleagues showed that miR-203 secreted by sEVs from pancreatic cancer could inhibit cytokines producing by DC via downregulation of TLR4 expression [108]. Moreover, DC differentiation and activation are inhibited by lung cancer-derived sEVs via a decrease of surface marker expression (like CD80, MHC-II, and CD86) and an increase of CD11B and PD-L1 expression [109].

Polarization of macrophages towards the cancer-promoting M2 phase is stimulated by tumor cell-derived sEVs: breast cancer-derived sEVs induce M2 polarization of macrophage via glycoprotein 130/STAT3 signaling, resulting in IL6 secretion [110]. sEVs, by releasing EMT factors such as HIFIα AND HIF2α, can promote migration, invasion, and EMT of cancer cells inducing macrophages to the M2 phenotype; this is what happens, for example, in hypoxic conditions by pancreatic cancer cell-derived sEVs [111]. Colon cancer cells mutant for p53 have been shown to release miR-1246-enriched sEVs, which trigger the differentiation of neighboring macrophages in macrophage M2by increasing TGFβ activity [112]. Breast cancer cell-derived sEVs are able to transfer EGFR in host macrophages which consequently reduce their production of type I interferons, thereby suppressing innate antiviral immunity and potentially inducing patients with cancer to become immunocompromised [113]. Gastric cancer-derived sEVs were shown to increase tumor-associated macrophages and impaired CD8+ T cell function via IL-10 (Zhang L et al. (2019)), while glioblastoma-derived sEVs skewed the monocytes toward the M2 phenotype, inducing activation of STAT3 or phosphorylation of p70S6 kinase and ERK1/2 [114]. Xiao reported that oral squamous cell carcinomas-derived sEVs polarize macrophages to the M1-like phenotype, transferring TSP1. MacrophagesM1-like phenotypes were activated through P38 MAPK, AKT, and SAPK/JNK signaling at the early phase and showed a pro-tumorigenic activity, promoting migration and proliferation of cancer cells [115].

**Figure 3:**
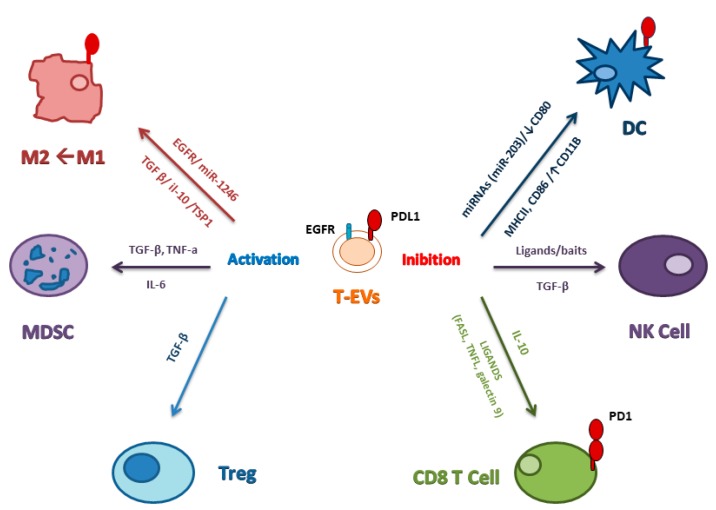
Immunity and extracellular vesicles in cancer. Schematic view of how T-sEVs repress the function of NK, T, and dendritic cells (DC) and activate the populations of myeloid-derived suppressive cells (MDSCs), regulatory T cells (Treg) while skewing macrophage function toward the M2 phenotype. PD-L1 packaged in T-sEVs is transferred to dendritic cells or macrophages which then block T cell function.

Therefore, sEVs can exert immunosuppressive but also immunoactive functions: the immune inhibition mainly depends on their capacity to carry proteins, ligands, and miRNAs against the activation of cytotoxic T cells or to promote the proliferation of immune-suppressive cells, while the immune activation mainly depends on antigen presentation by the sEVs surface. Understanding the main mechanisms regulating both functions will strongly contribute to the efforts to utilize sEVs in cancer treatment (Figure 3).

## 7. Metabolism of Immune Cells in Cancer and Relationship with EVs

Immune cells possess distinct metabolic programs compared to other cells. The function and the differentiation of immune cells are linked to their metabolism [116]. For example, naïve T cells differentiating into activated effector cells need much more energy and biosynthetic substrates and for this reason, cells switch on specific metabolic pathways [116]. The main players of metabolic pathways, as well as their metabolites, can directly induce immune cells activation. Naïve T cells can reprogram their metabolic pathway by the Warburg effect phenomenon using glucose for glycolysis [117]. Metabolites produced by glycolysis can also have effects on transcription, translation, and in protein activity in the immune system. After the activation of naïve T cells through TCR stimulation, there is a switch of metabolic programs from fatty acid β-oxidation and pyruvate oxidation via the TCA cycle to aerobic glycolysis. Moreover, MYC gene increases the level of glucose transporters such as GLUT-1 [118]. When the immune T cells do not express GLUT1, the glucose intake is lower and the proliferation and activation of effector T cells is prevented, and naïve T cells differentiate in Treg cells [119]. In ovarian cancer, effector T cells regulate amino acid metabolism of tumor cells, abrogating platinum resistance. In breast cancer, lactated-activated macrophages induce aerobic glycolysis by the CCL5-CCR5 axis and by Gpr132 [120,121]. Moreover, tumor-derived lactic acid can induce functional polarization of tumor-associated macrophages. Sukumar and colleagues reported that inhibiting the glycolytic pathway with 2-Deoxy-D-glucose during in vitro T cell activation results in higher and persistent anti-tumor activity mediated by memory-like CD8+ T cells [122]. On the other hand, the inhibition of OXPHOS has been shown to improve antitumor drug response by improving antitumor immunity: effector T cells with antitumor activity have been reported to have a glycolytic metabolism while immunosuppressive cells such as TREG have been shown to depend on OXPHOS for their power [123,124].

Overall, understanding the metabolic changes of immune cells can contribute to the improvement of immunotherapy strategies to enhance the immune responses against tumors. Recently, Fei Chen showed that tumor-associated macrophages enhance aerobic glycolysis and apoptotic resistance of breast cancer cells via EVs transfer of lncRNA HISLA [125]. Overall, these data provide the foundation of a possible relationship between the EVs released by tumor cells and the induction of changes in immune cell metabolism. Knowledge of these mechanisms is essential to understand how to correctly exploit the immune system as a tool to defeat cancer.

## 8. Cytokines and Extracellular Vesicles: Role in Cell Communication within Tumors

Most of the literature on the cross-role of extracellular vesicles and cytokines in cell communication within tumors is focused on sEVs, whose importance is progressively emerging (Table 3).

Immune and inflammatory cells can recognize the intracellular environment of cancer cells thanks to tumor-derived sEVs, which can stimulate secretion of growth factors and proinflammatory cytokines in recipient cells. Indeed, tumor-derived sEVs can stimulate a significant release of various cytokines, including IL-6, TNF, and TGF-β by CD14+ monocytes from healthy donors and can promote suppression of their functions as well as the proliferation of T cells [127].

Hood et al. showed how melanoma-derived sEVs could contribute to angiogenesis and metastasis by driving the secretion of proinflammatory cytokines (IL-1, FGF, GM-CSF, TNF, and VEGF) and angiogenic factors in endothelial cells [128]. Released sEVs can be loaded with proinflammatory cytokines (TNF, IL-6, and proteinases MMP2, MMP9), as shown in prostate cancer cells, increasing invasiveness and metastasis [126].

sEVs need TLR signaling to mediate tumor-promoting inflammation: sEVs released from lung cancer cells carry two types of miRNAs (miR-21 and miR-29a) that can bind to human TLR8 and murine TLR7, inducing the activation of NFκB signals and promoting tumor growth and metastasis by secretion of pro-inflammatory cytokines [127]. Similarly, breast cancer-derived sEVs induce NFκB activation via TLR2 in macrophages resulting in upregulation of IL-6, TNF-α, G-CSF, and CCL2 [131].

It is important to emphasize that sEVs may also contain stress proteins. For example, the interaction between tumor-derived sEVs and myeloid-derived suppressor cells (MDSC) is mediated by sEVs-membrane-associated Hsp72 and TLR2, which are responsible for MDSC activation. This binding results in STAT3 activation and autocrine production of IL-6 [132]. Moreover, HSP70 expressed on the surface of lung cancer cell line-derived sEVs activate NFκB signaling through TLR2 on mesenchymal stem cells and this binding induces a release of proinflammatory cytokines and chemokines, such as IL-8, IL-6, MCP [133]. Moreover, Hsp90 carried by sEVs can promote metastasis and enhance invasiveness of cancer cells by activating MMP2 and plasmin [127].

It is also well known that tumor-derived sEVs can stimulate the death of activated T-cells. For example, sEVs can express death ligands TRAIL and FasL, carrying immune cells to apoptosis, as in the case of ovarian cancer [127]. Moreover, MHCII-FasL sEVs produced by human B cell-derived lymphoblastoid cell lines can induce antigen-specific apoptosis in CD4+ T cells [134].

A recent study [129] showed how sEVs generated by acute myeloid leukemia cells trigger bone marrow stromal cells (BMSCs) to generate and secrete IL-8, which promotes the development of leukemia chemotherapy resistance. It was also reported that chronic myeloid leukemia cells release sEVs that stimulate IL-8 secretion by stromal cells by inducing expression of Snail that promotes the growth and invasiveness of leukemic cells [130].

## 9. Conclusions

In conclusion, cancer and tumor-associated stromal cells comprise an heterogeneous population of cells strongly inter-related one to each other, likely through multiple mechanisms in which EVs seem to play a pivotal, and still unknown, role. Thus, intratumor heterogeneity appears to be regulated not only by intrinsic factors (such as biomarker expression, genotype, epigenetic phenotype, metabolism, hypoxic state, and stage of differentiation of cells) but also by extrinsic factors related to tumor microenvironment such as cytokines, chemokine, growth factors, and EVs [135]. The discovery and characterization of EVs have highlighted a completely “new form” of intercellular communication and they appear to orchestrate many events related to cancer progression, invasion, and metastasis. A better knowledge of the molecular mechanisms triggered and/or regulated by EVs in relation to cancer-related metabolism and to the immune system is of particular relevance in the context of building new drug therapies to personalize the cure of cancer patients. Overall, a better knowledge of the messages carried out from both normal and cancer cells by EVs is of primary importance since it could provide a strategy against all rescue systems that cancer cells or tumor-surrounding cells activate under therapeutic pressure.

## Figures and Tables

**Table 1:** Major metabolic chemical intermediaries involved in communication between stromal and cancer cells and a synthetic view of their effects on cancer cells.

Producing Cells	Chemical Intermediaries	Effects on Cancer Cells	References
**CAFs**	M2-type pyruvate kinase; lactate dehydrogenase A; peroxiredoxin 1; atalase	Growth, proliferation, therapeutic resistance	[29,31,32,33,34]
**CAAs**	IGFBP-2; IL-6, IL-1β, IL-8	Matrix remodeling, invasion and survival, EMT, migration, metastasis, energy source	[38,39,40]
**Immune cells: MDSCs, TAMs, Tregs, TADCs, N1 neutrophils**	PD-L1, adenosine, lactate	Initiation, progression and metastasis, immunosuppression and angiogenesis	[42]

CAFs = cancer-associated fibroblasts; CAAs = Cancer-associated adipocytes; MDSCs = myeloid-derived suppressor cells; TAMs = tumor-associated macrophages; Tregs = regulatory T cells; TADCs= tumor-associated dendritic cells.

**Table 2:** Major microRNAs involved in fibroblasts conversion to CAFs.

microRNA	Reported Effects	References
**miR-155**	Pancreatic cancer cells reprogram normal adjacent fibroblasts into CAF by secreted mEVs containing miR-155.	[56]
**miR-9**	Breast cancer cells transfer miR-9 via sEVs and it affects the properties of human breast fibroblasts, enhancing the switch to CAF phenotype.	[57]
**miR-1247-3p**	Tumor-derived sEVs miR-1247-3p converts fibroblasts to CAFs by decreasing B4GALT3 and activation of β1-integrin–NF-κB signaling pathway in lung pre-metastatic niche from liver cancer.	[58]
**miR-27a**	miR-27a in sEVs derived from gastric cancer cells functions as an oncogene inducing the reprogramming of fibroblasts into CAFs	[59]
**miR-10b**	Colorectal cancer cells-derived sEVs transfer miR-10b that regulates fibroblasts via the PI3K/Akt pathway.	[60]
**miR-1249-5p, miR6737-5p, miR-6819-5p**	miR-1249-5p, miR-6737-5p and miR-6819-5p inhibition in fibroblasts can restore TP53 expression that is down-regulated in CAFs.	[61]
**miR-125b**	miR-125b is transferred through sEVs from breast cancer cells to normal fibroblasts and contributes to their switch to CAFs phenotype.	[62]
**miR142-3p**	sEVs-miR-142-3p from lung adenocarcinoma cells promotes CAFs phenotype in lung fibroblasts.	[63]

**Table 3:** Role of principal cytokines carried by sEVs and their role in cell communication within a tumor.

Source	Cytokines	Functions	References
**EVs**	IL-6	Increases invasiveness and metastasis of cancer cells.	[126]
**Cells stimulated by EVs**	IL-6	Suppresses functions and proliferation of T cells;	[38,39,40,127]
		modulates stromal cells function, migration and EMT;	
		increases tumor size and formation of connections.	
	TNF-α and IL-1-β	Regulate expression of MCP1 (CCL2), IL-8 (CXCL8) and RANTES (CCL6) in breast cancer;	[89,120,121,126,127,128]
		induce cell death in immune cells;	
		increase prostate cancer cell invasiveness;	
		promote angiogenesis in endothelial cells;	
		increase invasiveness and metastasis.	
	FGF, GM-CSF and VEGF	Promote angiogenesis in endothelial cells.	[128]
	IL-8	Promotes growth and invasiveness of leukaemic cells; promotes drug resistance.	[129,130]
